# Assessing the Gap Between Women's Expectations and Perceptions of the Quality of Intrapartum Care in Jordan: A Two‐Stage Study Using the SERVQUAL Model

**DOI:** 10.1111/hex.14103

**Published:** 2024-06-14

**Authors:** Heba Hijazi, Nabeel Al‐Yateem, Rabah Al abdi, Wegdan Baniissa, Mohamad Alameddine, Alham Al‐Sharman, Alounoud AlMarzooqi, Muhammad Arsyad Subu, Fatma Refaat Ahmed, Ahmed Hossain, Amer Sindiani, Yaseen Hayajneh

**Affiliations:** ^1^ Department of Health Care Management, College of Health Sciences University of Sharjah Sharjah UAE; ^2^ Department of Health Management and Policy, Faculty of Medicine Jordan University of Science and Technology Irbid Jordan; ^3^ Nursing Department, College of Health Sciences University of Sharjah Sharjah UAE; ^4^ Department of Electrical, Computer, and Biomedical Engineering, College of Engineering Abu Dhabi University Abu Dhabi UAE; ^5^ Department of Biomedical Engineering, Faculty of Engineering Jordan University of Science and Technology Irbid Jordan; ^6^ Department of Physiotherapy, College of Health Sciences University of Sharjah Sharjah UAE; ^7^ Rehabilitation Sciences Department, Faculty of Applied Medical Sciences Jordan University of Science and Technology Irbid Jordan; ^8^ Critical Care and Emergency Nursing Department, Faculty of Nursing Alexandria University Alexandria Egypt; ^9^ Department of Obstetrics and Gynsecology, Faculty of Medicine Jordan University of Science and Technology Irbid Jordan; ^10^ Ancell School of Business Western Connecticut State University Danbury Connecticut USA

**Keywords:** intrapartum care, labour and birth care, quality of care, SERVQUAL scale, women health

## Abstract

**Introduction:**

Although Jordan has made significant progress toward expanding the utilization of facility‐based intrapartum care, prior research highlights that poor service quality is still persistent. This study aimed to identify quality gaps between women's expectations and perceptions of the actual intrapartum care received, while exploring the contributing factors.

**Methods:**

Utilizing a pre–post design, quality gaps in intrapartum care were assessed among 959 women pre‐ and postchildbirth at a prominent tertiary hospital in northern Jordan. Data were gathered using the SERVQUAL scale, measuring service quality across reliability, responsiveness, tangibles, assurance, and empathy dimensions.

**Results:**

The overall mean gap score between women's expectations and perceptions of the quality of intrapartum care was −0.60 (±0.56). The lowest and highest mean gap scores were found to be related to tangibles and assurance dimensions, −0.24 (±0.39) and −0.88 (±0.35), respectively. Significant negative quality gaps were identified in the dimensions of assurance, empathy, and responsiveness, as well as overall service quality (*p* < 0.001). The MLR analyses highlighted education (*β* = 0.61), mode of birth (*β* = −0.60), admission timing (*β* = −0.41), continuity of midwifery care (*β* = −0.43), physician's gender (*β* = −0.62), active labour duration (*β* = 0.37), and pain management (*β* = −0.33) to be the key determinants of the overall quality gap in intrapartum care.

**Conclusion:**

Our findings underscore the importance of fostering a labour environment that prioritizes enhancing caregivers' empathetic, reassuring, and responsive skills to minimize service quality gaps and enhance the overall childbirth experience for women in Jordan.

**Patient or Public Contribution:**

This paper is a collaborative effort involving women with lived experiences of childbirth, midwives, and obstetrics and gynaecologist physicians. The original idea, conceptualization, data generation, and coproduction, including manuscript editing, were shaped by the valuable contributions of stakeholders with unique perspectives on intrapartum care in Jordan.

## 1 Introduction

Millions of women in low‐ and middle‐income countries continue to experience life‐threatening conditions due to the poor and inadequate care and support provided during the intrapartum and immediate postnatal periods [1, 2]. In response, governments, policy makers and regulators have advocated optimizing the quality of intrapartum care as a key strategy to significantly reduce maternal and infant mortality rates, including preventable causes of deaths [3, 4]. In 2018, the World Health Organization (WHO) published a set of recommendations for intrapartum care as a crucial priority for ensuring a positive childbirth experience for women and their babies. According to WHO's guidelines, intrapartum care is defined as the comprehensive care provided to a woman during the entire process of childbirth, covering a wide range of elements related to all stages of labour and immediate care for the woman and the baby after birth [5]. These elements include providing respectful and responsive maternity care, offering emotional and physical support, ensuring effective pain management, facilitating informed decision‐making, creating a safe and hygienic labouring environment, regularly monitoring labour progress, and maintaining continuity of care [5, 6].

Over the last few decades, numerous tools have been developed to evaluate service quality. However, the service quality (SERVQUAL) scale, developed by Parasuraman et al. [7], is still considered one of the most widely used, valid, and reliable tools for measuring service quality across the dimensions of reliability, responsiveness, tangibles, assurance, and empathy [8]. This instrument has been extensively applied in healthcare settings to identify areas where healthcare providers may fall short of patients' expectations, explore areas for improvement in service delivery, and track changes in service quality over time [9].

Considering that most service users may lack sufficient information to evaluate technical aspects of service quality, such as the accuracy of medical diagnoses and the effectiveness of treatment, the SERVQUAL model primarily emphasizes the functional quality aspect [10, 11]. This aspect focuses on internal service procedures, encompassing provider empathy, service delivery reliability, and staff responsiveness [10]. Further, the model places significant emphasis on the concept of the ‘quality gap’, which represents the discrepancy between users' preservice expectations and their perceptions of actual service experience [12].

Quality gaps in intrapartum care often arise when the services, communication, and support provided during childbirth do not fully align with the mothers' expectations, leading to potential gaps in their overall satisfaction with the care received [13]. Several studies have indicated that experiencing a quality gap in intrapartum care may lead to various adverse consequences for women, such as an increased risk of developing postpartum depression and anxiety [14, 15], posttraumatic stress symptoms [16], challenges in mother–infant attachment [17], breastfeeding difficulties [18], longer intervals between pregnancies [19], and fears of subsequent births [2, 15].

Until the late 1970s and 1980s, home deliveries were prevalent for Middle Eastern women, including those in Jordan [20]. Traditional childbirth assistance during this period came from respected community women known as Dayas [20]. Within the initiatives undertaken by the Jordanian Ministry of Health to enhance the health of mothers and children, various policies have been implemented over the last few decades. There has been a particular emphasis on the importance of skilled birth attendance and hospital births, resulting in 98% of births currently occurring in hospitals [3]. While progress has been made in expanding facility‐based intrapartum care in Jordan, substantial efforts are still needed to address critical barriers and ensure quality care. Research has reported that poor‐quality intrapartum care is widespread in the country due to several reasons [14, 18, 21]. These include neglecting women's needs and preferences, encountering tangible/physical noncaring behaviours, experiencing feelings of disrespect and abandonment, inadequate communication and information sharing, and the exclusion of women from decision‐making processes [18, 21].

In the Jordanian context, prior research has also underscored a prevalent practice where having a companion during labour is generally not permitted in most hospitals [20, 22, 23]. This restriction, allowing only the husband and solely in specific cases, has prompted some women to actively seek alternatives, often turning to private hospitals that cater to their distinct needs. Within these private hospital settings, women have reported a heightened sense of control over various elements, including maintaining their privacy and having options in pain management, fostering a desire for a more personalized birthing experience [20].

Currently, data on the quality gap in childbirth service delivery at Jordanian health facilities is limited, with much research focusing on women's general satisfaction with services being provided. Therefore, our study aimed to measure the quality gaps and identify the service quality dimensions exhibiting the most significant disparities between women's expectations and perceptions of the quality of intrapartum care in North Jordan, as well as explore the contributing factors to the service quality gaps.

## 2 Methods

### 2.1 Study Design and Procedures

Using a facility‐based pre–post design, our study employed a two‐stage methodology to assess the gaps between women's expectations and perceptions of the quality of intrapartum care.

#### 2.1.1 Stage 1

To assess women's expectations of service quality before childbirth, a sample of pregnant women was approached via face‐to‐face communication at obstetrical and gynaecological clinics at a leading tertiary hospital in northern Jordan. Using a convenience sampling technique, a total of 1696 pregnant women were contacted and screened for participation eligibility. Exclusion criteria consisted of being younger than 18 or older than 45 years of age, being in the first or second trimester of pregnancy (less than 28 weeks of gestation), planning to deliver at a different facility, or refusing to participate in either of the study's two stages. Our study specifically focused on women in the latter stages of pregnancy to capture representative intrapartum care experiences closer to their delivery dates and ensure a more homogeneous group of participants. Of the 1696 women approached, 623 were excluded based on previous criteria, resulting in a sample size of 1073 for the first stage of the study. To ensure uniformity in data collection throughout both study stages, a single skilled and well‐trained female interviewer conducted the interviews. The data collection for Stage 1 took place from June 2018 to September 2019.

#### 2.1.2 Stage 2

During Stage 1, the interviewer recorded participants' phone numbers and expected delivery dates for follow‐up. To complete Stage 2, all mothers who participated in the first stage were contacted via telephone within the first 3 months postpartum to retrospectively evaluate their perceptions of actual childbirth experiences. While follow‐up appointments could be set within the first month, actual interviews were deliberately postponed during this period to accommodate mothers' ongoing emotional and physical changes, ensuring they had sufficient time for recovery. Accordingly, the interviews took place during the second and third months postpartum. The chosen timeframe was considered optimal for mothers to assimilate and reflect on their experiences while minimizing recall bias [1, 24], and it also focuses on a more homogeneous group of participants, thereby contributing to the enhanced accuracy of the results. The transition to phone follow‐up in the second stage was essential to prevent additional burden on postpartum women, enhance flexibility, and encourage increased participation.

**Figure 1:**
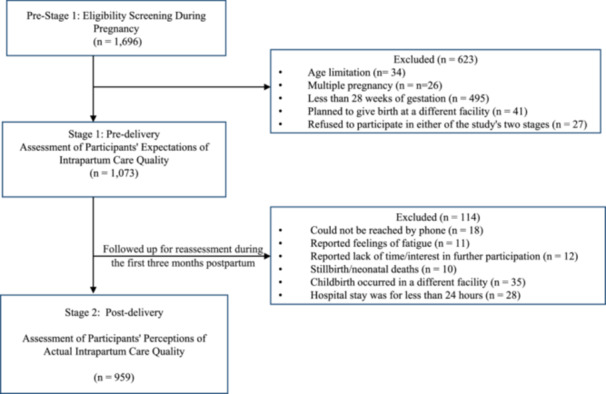
Participant flowchart.

To confirm eligibility for Stage 2, the interviewer first asked the participant a screening question to ensure that she delivered a singleton live infant at the same hospital where her expectations were assessed earlier, and that she stayed in the hospital for at least 24 h. Of the 1073 mothers who participated in Stage 1, 114 (10.6%) were excluded from the final analysis due to stillbirth/neonatal deaths, contact difficulties, giving birth in a different facility, less‐than‐24‐h hospital stay, or due to reported feelings of fatigue, lack of time, or disinterest in further participation. As a result, the final sample comprised 959 mothers who met the inclusion criteria of the second stage and had completed the two stages of the study successfully. Figure 1 presents a comprehensive breakdown of the participation rates for each stage of the study.

### 2.2 Study Tool and Measures

Data was gathered using the SERVQUAL tool, which consisted of 22 items rated on a 5‐point Likert scale with scores ranging from 1 (strongly disagree) to 5 (strongly agree). Higher scores on each dimension item indicate higher levels of service quality. The tool measured five dimensions of service quality, including tangibles or physical services (four items), reliability of services (four items), and responsiveness (four items), assurance (four items), and empathy of service providers (five items).

In our study, the SERVQUAL scale was administered twice; once per stage. During the initial stage, participants were questioned about their sociodemographic status (i.e., age, education, parity, place of residence, and income), as well as their expectations for the 22 items in the SERVQUAL tool (i.e., do you expect the use of up‐to‐date equipment and technology?; and do you expect to receive services at the times promised?).

Following participants for the first 3 months postpartum, we conducted a repeated administration of the SERVQUAL survey, where participants provided us with additional information about the time and mode of delivery, the intensity of pain during labour, the availability of pain‐relief options, the gender of the primary obstetrician, and the duration of active labour. Women were considered as being in active labour phase when there is the presence of regular uterine contractions with cervical dilatation of 4 cm or more [25]. We defined the duration of active labour as the time from the onset of the active phase to the delivery of the baby. As a benchmark for comparison, participants were requested to assess their perceptions of actual intrapartum care they had received using the same set of 22 items used in the first stage (i.e., did you encounter the use of up‐to‐date equipment and technology; and did you receive services at the times promised?).

The SERVQUAL survey has been extensively utilized to assess the quality of health services across various populations, including Romanian [26], Turkish [27], Bangladeshi [28], and Iranian [8]. Further, prior research has suggested that the SERVQUAL scale is a suitable instrument for measuring service quality in the Arabian context [29, 30], and across several healthcare settings, including hospitals, clinics, and primary healthcare centres [31, 32]. However, to align with the context of our study, we adapted the SERVQUAL survey to enhance its relevance and applicability within the labouring environment. Accordingly, we conducted further tests to assess the validity and reliability of the modified survey.

To maintain the intended meaning of the scale items, standard forward and backward translation protocols were applied during the translation process. Furthermore, we sought feedback from a panel of experts in obstetrics and gynaecologist, nursing, and midwifery to establish content validity. This panel thoroughly reviewed the survey items and individually evaluated and rated each item, considering its clarity, relevance, and appropriateness to the childbirth experience. They utilized a rating scale ranging from 1 (not relevant) to 4 (very relevant). The level of content validity was determined among the experts by calculating the content validation ratio (CVR). The CVR yielded an average of 0.89, demonstrating a strong content validity for the Arabic version of the survey [33].

Moreover, we conducted an exploratory factor analysis (EFA) with oblique promax rotation to establish the construct validity of the survey's Arabic version and identify underlying dimensions within the data set. The Kaiser–Meyer–Olkin (KMO = 0.853, *p* < 0.001) test for sampling adequacy and the Barlett test results (*χ* ^2^ = 1902.58, *p* < 0.001) for homogeneity of variances confirmed that the assumptions for EFA were satisfied. Using eigenvalues greater than one, five factors were identified, explaining 66.73% of the variance. Table 1 displays the factor loadings for each survey item obtained from the analysis. All included items exhibited loadings greater than 0.5 on their respective factors, indicating a satisfactory purification of constructs [34].

**Table 1:** Factor structure of the Arabic version of the SERVQUAL.

No.	Scale item	Rotated components				
		1	2	3	4	5
Dimension 1: Tangibles						
1.	Using up‐to‐date equipment and technology	0.13	0.04	0.03	**0.79**	0.07
2.	Having visually appealing physical facilities	0.07	−0.12	−0.06	**0.84**	0.11
3.	Maintaining a clean & comfortable labouring environment in the hospital (i.e., bed, delivery room, toilets)	−0.03	0.05	0.12	**0.89**	−0.08
4.	Having labour and delivery staff who are neat & well‐dressed	0.10	−0.04	0.09	**0.86**	0.13
Dimension 2: Reliability						
5.	Exhibiting consistency and dependability in solving patients' problems	0.04	0.11	**0.85**	0.09	0.07
6.	Giving periodic updates on status and progress of labour services	0.09	0.03	**0.74**	0.13	0.08
7.	Ensuring error‐free & fast retrieval of documents	0.03	0.07	**0.83**	0.05	0.13
8.	Performing the services right at the first time	0.12	0.08	**0.88**	0.06	0.09
9.	Carrying out services at promised time	0.07	0.11	**0.82**	0.12	0.10
Dimension 3: Responsiveness						
10.	Providing prompt performance of medical & nonmedical services	0.11	**0.89**	0.05	−0.08	0.14
11.	Telling when services will be performed	0.05	**0.84**	−0.08	0.13	0.08
12.	Demonstrating a willingness to help patients	0.09	**0.88**	0.14	−0.11	0.04
13.	Attending patient's calls promptly	0.12	**0.87**	−0.10	0.05	0.09
Dimension 4: Assurance						
14.	Making patients feel safe and secure during interactions with the labour and delivery staff	**0.85**	0.11	0.04	−0.03	0.10
15.	Having a trustworthy labour and delivery staff	**0.83**	0.07	0.013	−0.09	0.14
16.	Dealing with patients politely	**0.89**	−0.09	0.04	0.11	0.07
17.	Providing the patient with adequate support during labour	**0.81**	0.13	0.08	0.15	0.05
Dimension 5: Empathy						
18.	Addressing patients' specific preferences, concerns, and emotions	0.09	0.13	0.08	−0.03	**0.88**
19.	Understanding specific needs of patients	0.11	0.09	0.04	0.05	**0.87**
20.	Having patients' best interest at heart	0.13	0.10	0.03	−0.03	**0.74**
21.	Having operating hours convenient for the patient	0.04	0.12	0.12	0.12	**0.83**
22	Customizing care to meet individual patients' needs and characteristics	0.09	0.13	0.04	−0.07	**0.84**
Variance explained %		17.13	15.52	13.83	11.84	8.41
Cumulative percentage of variance (%)		17.13	32.65	46.48	58.32	66.73
McDonald's *ω*		0.81	0.84	0.78	0.75	0.80

*Note:* All significant loadings are in bold.

To ensure survey reliability, we conducted test‐retest and internal consistency analyses. A subgroup of participants (*n* = 30) completed the survey twice, 2 weeks apart. The test‐retest correlation coefficients showed a high correlation, with a value of 0.87 for the total scale and a range from 0.73 to 0.85 for the subscales, indicating a strong and consistent relationship between the measurements taken at different timepoints. McDonald's *ω* was also employed to assess internal consistency. Within the range of 0.75 to 0.84, McDonald's *ω* indicated acceptable to good reliability for the SERVQUAL's five dimensions [35]. The overall scale had a McDonald's *ω* of 0.81.

### 2.3 Data Analysis

The Statistical Package for the Social Sciences (SPSS) version 21 was utilized for data analysis. Descriptive analysis determined counts, percentages, mean scores, and standard deviations of study variables. To calculate the mean score for each quality dimension, the subtotal item scores were summed and divided by the number of items in the relevant dimension. In our analysis, we quantified the service quality gaps by subtracting the postdelivery perception scores from the pre‐delivery expectation scores. Wilcoxon test was applied to measure the significance of these gaps. Where perception scores exceed expectations, a positive quality gap arises, indicating that the service is excelling. Conversely, when a negative gap results, this signifies areas where service quality may not meet expectations, highlighting the need for improvement.

All quality dimensions that showed statistically significant quality gaps between mothers' expectations and perceptions were further tested using multiple linear regression (MLR) analyses to explore the contributing factors to each of the identified gaps. Variables with a *p* value below 0.05 were considered statistically significant.

### 2.4 Ethical Statement

The Institutional Review Board (IRB) of the Jordan University of Science and Technology and the Ethics Committee at the Kind Abdullah University Hospital (KAUH) approved the study protocol (# 20160089). Informed consent was obtained from all women, while ensuring their voluntary and confidential participation in both stages of the study. The study was conducted in accordance with the Declaration of Helsinki.

## 3 Results

### 3.1 Descriptive Statistics

Table 2 presents the summary statistics of the participants. The recruited women had ages ranging from 18 to 45 years, with a median age of 30 years. On average, the participants had completed 12 years of education. The sample predominantly composed of multiparous women (76.3%), housewives (71.6%), and urban residents (62.7%), with 93.7% receiving the WHO's newly recommended minimum of at least eight antenatal care visits [36].

**Table 2:** Characteristics of the participants (*N* = 959).

Variable	Frequency	(%)
Age (years)		
18–24	267	27.8
25–31	371	38.7
32–38	243	25.3
39–45	78	8.2
Education (years)		
≤6	25	2.6
7–9	159	16.6
10–12	464	48.4
>12	311	32.4
Employment status		
Housewives	687	71.6
Employed	272	28.4
Financial resources		
Inadequate	632	65.9
Adequate	327	34.1
Area of residence		
Rural	358	37.3
Urban	601	62.7
Parity		
First child	227	23.7
2–3 child	458	47.8
>3 children	274	28.5
No. of antenatal visits		
<8	60	6.3
≥8	899	93.7
Mode of birth		
Spontaneous vaginal delivery	496	51.7
Instrument delivery	72	7.5
Caesarean section	391	40.8
Presence of delivery complications		
No complications	459	47.9
Intrapartum haemorrhage	198	20.6
Perineal tear	157	16.4
Retained placenta	98	10.2
Shoulder dystocia	47	4.9
Length of active phase of labour (h)		
<6	262	27.3
6–8	485	50.6
>8	212	22.1
Infant gender		
Male	455	47.4
Female	504	52.6
Self‐reported health conditions at discharge		
Poor	213	22.2
Good	553	57.7
Excellent	193	20.1
Admission time		
Late at night (12 AM–5:59 AM)	87	9.1
Morning (6:00 AM–11:59 AM)	444	46.3
Afternoon (12:00 PM–5:59 PM)	275	28.7
Evening (6:00 PM–11:59 PM)	153	15.9

Out of the 959 women who participated in our study, 46.3% were admitted in the morning between 6:00 AM and 11:59 AM, 51.7% had spontaneous vaginal deliveries, 50.6% experienced an active labour phase lasting 6–8 h, and 52.6% of the infants were females. Approximately 48% of the sample experienced a low‐risk pregnancy with no reported complications during delivery. Intrapartum haemorrhage (20.6%) and perineal tear (16.4%) were the two most common delivery‐related complications among participants, respectively. Furthermore, 57.7% of the participants rated their health conditions at discharge as good, indicating a satisfactory level of well‐being.

### 3.2 Numerical Summary of SERVQUAL Dimensions

Table 3 displays the mean scores for women's expectations, perceptions, and gaps in intrapartum care quality dimensions. On the used 5‐point Likert scale, the mean scores for predelivery expectations ranged from 4.13 (item 8: Performing the services right at the first time) to 4.78 (item 18: Addressing patients' specific preferences, concerns, and emotions). Among the five quality dimensions, the empathy dimension had the highest expectation score with a mean score of 4.72, while the reliability dimension exhibited the lowest expectation score with a mean score of 4.51. The total mean score for women's expectations regarding the quality of intrapartum care provision was 4.69.

**Table 3:** Mean scores for women's expectations, perceptions, and quality gaps in intrapartum care dimensions.

Scale item	Mean perception score (±SD)	Mean expectation scores (±SD)	Mean quality gap scores	*p* value^a^
Dimension 1: Tangibles	4.28 (0.51)	4.52 (0.67)	−0.24	0.381
1. Using up‐to‐date equipment and technology	4.57 (0.93)	4.20 (0.68)	0.37	<0.001
2. Having visually appealing physical facilities	4.23 (0.74)	4.35 (0.55)	−0.12	0.309
3. Maintaining a clean & comfortable labouring environment in the hospital (i.e., bed, delivery room, toilets)	4.36 (0.75)	4.56 (0.87)	−0.20	0.018
4. Having labour and delivery staff who are neat & well‐dressed	4.55 (0.89)	4.59 (0.73)	−0.09	0.533
Dimension 2: Reliability	4.20 (0.64)	4.51 (0.46)	−0.31	0.261
5. Exhibiting consistency and dependability in solving patients' problems	4.56 (0.71)	4.15 (0.65)	0.41	0.516
6. Giving periodic updates on status and progress of labour services	4.13 (0.69)	4.53 (0.83)	−0.40	<0.001
7. Ensuring error‐free & fast retrieval of documents	4.56 (0.74)	4.67 (0.66)	−0.11	0.151
8. Performing the services right at the first time	3.88 (0.61)	4.13 (0.74)	−0.25	<0.001
9. Carrying out services at promised time	4.22 (0.65)	4.55 (0.76)	−0.33	0.011
Dimension 3: Responsiveness	3.91 (0.57)	4.66 (0.66)	−0.75	<0.001
10. Providing prompt performance of medical & nonmedical services	3.85 (0.76)	4.57 (0.72)	−0.72	<0.001
11. Telling when services will be performed	3.76 (0.80)	4.68 (0.70)	−0.92	<0.001
12. Demonstrating a willingness to help patients	3.92 (0.78)	4.56 (0.71)	−0.64	<0.001
13. Attending patient's calls promptly	4.12 (0.70)	4.73 (0.73)	−0.61	<0.001
Dimension 4: Assurance	3.83 (0.71)	4.71 (0.45)	−0.88	<0.001
14. Making patients feel safe and secure during interactions with the labour and delivery staff	3.96 (0.80)	4.74 (0.72)	−0.78	<0.001
15. Having a trustworthy labour and delivery staff	3.88 (0.83)	4.56 (0.72)	−0.68	<0.001
16. Dealing with patients politely	3.67 (0.76)	4.64 (0.69)	−0.97	<0.001
17. Providing the patient with adequate support during labour	3.85 (0.89)	4.77 (0.71)	−0.92	<0.001
Dimension 5: Empathy	3.90 (0.52)	4.72 (0.52)	−0.82	<0.001
18. Addressing patients' specific preferences, concerns, and emotions	3.85 (0.76)	4.78 (0.72)	−0.93	<0.001
19. Understanding specific needs of patients	3.76 (0.81)	4.69 (0.70)	−0.93	<0.001
20. Having patients' best interest at heart	3.90 (0.80)	4.76 (0.71)	−0.86	<0.001
21. Having operating hours convenient for the patient	4.44 (0.93)	4.37 (0.68)	0.07	0.025
22. Customizing care to meet individual patients' needs and characteristics	3.66 (1.06)	4.64 (0.69)	−0.98	<0.001
Overall service quality	4.09 (0.71)	4.69 (0.65)	−0.60	<0.001

^a^ Extracted using Wilcoxon test.

The mean scores of items related to women's postdelivery perceptions varied, ranging from 3.66 (item 22: Customizing care to meet individual patients' needs and characteristics) to 4.57 (item 1: Using modern and up‐to‐date equipment and technology). Among the five quality dimensions, the tangibles dimension had the highest perception score with a mean score of 4.28, while the assurance dimension displayed the lowest perception score with a mean score of 3.83. The overall mean score for women's perceptions of the quality of intrapartum care experienced was 4.09.

As per Table 3, the perception scores were consistently lower than the corresponding expectation scores for all SERVQUAL dimensions, indicating the presence of solely negative quality gaps in the intrapartum care provided. The Wilcoxon test results indicated statistically significant gaps in the dimensions of responsiveness, assurance, and empathy, as well as the overall quality (*p* < 0.001). Notably, the assurance dimension exhibited the highest negative quality gap, followed by the empathy and responsiveness dimensions, with mean score gaps of −0.88, −0.82, and −0.75, respectively. While the tangibles and reliability dimensions exhibited negative quality gaps with mean scores of −0.24 and −0.31, respectively, the Wilcoxon test results showed that these gaps were not statistically significant (*p* = 0.381, and *p* = 0.261, respectively). Overall, there was a statistically significant negative gap of −0.60 between the total mean scores of women's expectations and their perceptions of the actual quality of intrapartum care (*p* < 0.001).

### 3.3 Predictors of the Quality Gaps in Intrapartum Care

In our analysis, four MLR models were developed to explore the contributing factors to each of the previously identified significant negative gaps.

### 3.4 Responsiveness Gap

The coefficients estimated from the first MLR model identified women's age and education as significant predictors of the responsiveness gap. For every 1‐year increase in the woman's age and education, the responsiveness mean score gap increased by factors of 0.22 (95% confidence interval [95% CI]: 0.13–0.30), and 0.75 (95% CI: 0.69–0.83), respectively.

Our findings also demonstrated that mothers who underwent caesarean births (*β* = −0.65; 95% CI: −0.76 to −0.54), had morning admissions (*β* = −0.35; 95% CI: −0.51 to −0.24), received care from a familiar midwife (*β* = −0.45; 95% CI: −0.56 to −0.32), and experienced more than two prior labour admissions at the same hospital (*β* = −0.13; 95% CI: −0.21 to −0.08) showed significant decrease in the responsiveness mean score gap. In contrast, spending more than 8 h in the active phase of labour (*β* = 0.28; 95% CI: 0.15–0.42) was identified as a significant predictor correlated with increased mean gap score in the responsiveness dimension.

### 3.5 Assurance Gap

As per Table 4, the second model results indicated that the assurance gap increased with higher education levels (*β* = 0.78; 95% CI: 0.71–0.89) and higher‐than‐expected pain levels (*β* = 0.29; 95% CI: 0.17–0.41). In contrast, this gap decreased for those who had caesarean births (*β* = −0.44; 95% CI: −0.56 to −0.35), female obstetrician (*β* = −0.56; 95% CI: −0.67 to −0.45), and a familiar midwife present during their childbirth (*β* = −0.37; 95% CI: −0.45 to −0.35).

**Table 4:** Estimated MLR coefficients for the mean gap scores in intrapartum care quality dimensions.

Variable	Dimension			
	Responsiveness Model 1, *β* (95% CI)	Assurance Model 2, *β* (95% CI)	Empathy Model 3, *β* (95% CI)	Overall Quality Final Model, *β* (95% CI)
*Sociodemographic factors*				
Age in years	**0.22 (0.13–0.30)**	0.35 (−0.05 to 0.75)	0.28 (−0.12 **to** 0.68)	0.31 (−0.09 to 0.71)
Education in years	**0.75** (**0.69–0.83**)	**0.78** (**0.71–0.89**)	**0.55** (**0.42–0.61)**	**0.61** (**0.52–0.73)**
Employment status: Housewives (ref)				
Employed	0.11 (−0.19 to 0.34)	0.18 (−0.12 to 0.48)	0.22 (−0.08 to 0.52)	0.14 (−0.16 to 0.44)
Financial resources: Inadequate (ref)				
Adequate	0.19 (−0.11 to 0.49)	0.25 (−0.05 to 0.55)	0.30 (−0.04 to 0.64)	0.23 (−0.07 to 0.53)
Area of residence: Rural (ref)				
Urban	0.34 (−0.06 to 0.74)	0.41 (−0.02 to 0.82)	0.29 (−0.09 to 0.67)	0.37 (−0.03 to 0.77)
*Obstetric history*				
No. of antenatal visits: <8 (ref)				
≥8	0.24 (−0.14 to 0.59)	0.31 (−0.09 to 0.71)	0.27 (−0.13 to 0.67)	0.30 (−0.11 to 0.68)
Parity: First child (ref)				
2–3 child	−0.14 (−0.58 to 0.30)	−0.19 (−0.52 to 0.14)	−0.15 (−0.40 to 0.10)	−0.20 (−0.45 to 0.05)
>3 children	−0.26 (−0.65 to 0.21)	−0.18 (−0.50 to 0.14)	−0.21 (−0.47 to 0.05)	−0.25 (−0.54 to 0.04)
Mode of birth: Spontaneous vaginal delivery (ref)				
Instrument delivery	−0.21 (−0.61 to 0.16)	−0.30 (−0.70 to 0.10)	−0.27 (−0.67 to 0.13)	−0.35 (−0.85 to 0.15)
Caesarean section	**−0.65 (−0.76 to −0.54)**	**−0.44** (**−0.56 to −0.35)**	**−0.51** (**−0.68 to −0.44)**	**−0.60** (**−0.75 to −0.53)**
Duration of active phase of labour: <6 h (ref)				
6–8 h	0.09 (−0.31 to 0.49)	0.19 (−0.12 to 0.50)	0.14 (−0.07 to 0.35)	0.22 (−0.02 to 0.44)
>8 h	**0.28** (**0.15 to 0.42**)	0.30 (−0.08 to 0.68)	**0.43** (**0.31–0.57**)	**0.37** (**0.21–0.49**)
Presence of delivery complications: no complications (ref)				
Intrapartum haemorrhage	0.16 (−0.24 to 0.35)	0.28 (−0.12 to 0.68)	**0.39** (**0.23–0.52)**	0.22 (−0.15 to 0.59)
Perineal tear	0.23 (−0.17 to 0.49)	0.19 (−0.21 to 0.59)	0.17 (−0.23 to 0.57)	0.20 (−0.12 to 0.52)
Retained placenta	0.31 (−0.09 to 0.71)	29 (−0.11 to 0.69)	0.28 (−0.22 to 0.77)	0.32 (−0.04 to 0.68)
Shoulder dystocia	0.15 (−0.25 to 0.35)	27 (−0.13 to 0.67)	0.31 (−0.18 to 0.64)	0.26 (−0.08 to 0.60)
*Service‐related factors*				
Continuity of midwifery care: Unknow midwife (ref)				
Known midwife	**−0.45** (**−0.56 to −0.32)**	**−0.37** (**−0.45 to 0.35**)	−0.11 (−0.50 to 0.28)	**−0.43** (**−0.51 to −0.35)**
Physician's gender: Male (ref)				
Female	−0.17 (−0.45 to 0.11)	**−0.56** (**−0.67 to 0.45)**	**−0.67** (**−0.82 to −0.55)**	**−0.62** (**−0.77 to −0.49)**
Admission timing: Late at night [12 AM–5:59 AM] (ref)				
Morning [6:00 AM–11:59 AM]	**−0.35** (**−0.51 to −0.24)**	−0.28 (−0.68 to 0.12)	−0.31 (−0.71 to 0.09)	**−0.41** (**−0.52 to −0.30)**
Afternoon [12:00 PM–5:59 PM]	−0.20 (−0.48 to 0.08)	−0.25 (−0.65 to 0.15)	−0.36 (−0.83 to 0.11)	−0.38 (−0.73 to 0.10)
Evening [6:00 PM–11:59 PM]	−0.15 (−0.85 to 0.19)	−0.20 (−0.60 to 0.20)	−0.28 (−0.68 to 0.12)	−0.25 (−0.65 to 0.15)
History of previous hospitalization: No previous admission (ref)				
1–2 times	−0.23 (−0.54 to 0.11)	−0.31 (−0.79 to 0.19)	−0.28 (−0.62 to 0.08)	−0.21 (−0.55 to 0.16)
≥2 times	**−0.13** (**−0.21 to −0.08**)	−0.17 (−0.52 to 0.18)	−0.25 (−0.59 to 0.11)	−0.30 (−0.64 to 0.04)
*Pain assessment and management*				
Self‐reported health conditions at discharge: Poor (ref)				
Good	−0.44 (−1.06 to 0.17)	−0.33 (−0.74 to 0.10)	−0.41 (−1.04 to 0.17)	−0.39 (−0.70 to 0.08)
Excellent	−0.51 (−1.12 to 0.13)	−0.45 (−1.12 to 0.13)	−0.38 (−1.01 to 0.25)	−0.46 (−0.88 to 0.04)
Pain assessment during labour: Lower than expected (ref)				
Matched expectations	0.34 (−0.15 to 0.79)	0.13 (−0.11 to 0.27)	0.25 (−0.05 to 0.55)	0.30 (−0.10 to 0.70)
Higher than expected	0.50 (−0.09 to 1.09)	**0.29** (**0.17–0.41**)	**0.41** (**0.30–0.52**)	0.38 (−0.11 to 0.79)
Pain management provided by labour staff: Limited assistance (ref)				
Adequate assistance	−0.16 (−0.57 to 0.27)	−0.32 (−0.68 to 0.04)	−0.14 (−0.59 to 0.31)	−0.20 (−0.17 to 0.56)
Fully supported in managing pain	−0.25 (−0.67 to 0.17)	−0.28 (−0.61 to 0.05)	**−0.37** (**−0.45 to −0.26**)	**−0.33** (**−0.45 to −0.22**)
Model summary				
*R* ^2^	0.75	0.66	0.74	0.73
Adjusted *R* ^2^	0.71	0.60	0.69	0.70

*Note:* All significant associations are in bold.

### 3.6 Empathy Gap

In the third MLR model, our results revealed that for every 1‐year increase in the woman's education, the empathy gap increased by a factor of 0.55 (95% CI: 0.42–0.61). Additionally, mean gap scores in the empathy dimension significantly increased for mothers who experienced postpartum haemorrhage (PPH) (*β* = 0.39; 95% CI: 0.23–0.52), over 8‐h active labour phase (*β* = 0.43; 95% CI: 0.31–0.57), and higher‐than‐expected pain (*β* = 0.41; 95% CI: 0.30–0.52). Conversely, women who underwent caesarean births (*β* = −0.51; 95% CI: −0.68 to −0.44), perceived full support in managing pain (*β* = −0.37; 95% CI: −0.45 to −0.26), and had a female obstetrician (*β* = −0.67; 95% CI: −0.82 to −0.55) exhibited significant reductions in the empathy gap.

### 3.7 Overall Service Quality Gap

Considering the overall service quality, the final model results demonstrated that the overall intrapartum care quality gap increased for mothers who had more years of education (*β* = 0.61; 95% CI: 0.52–0.73) and experienced an active labour phase lasting over 8 h (*β* = 0.37; 95% CI: 21–0.49). However, overall quality gap reduced when mothers had morning admissions (*β* = −0.41; 95% CI: −0.52 to −0.30), caesarean births (*β* = −0.60; 95% CI: −0.75 to −0.53), a female obstetrician (*β* = −0.62; 95% CI: −0.77 to −0.49) accompanied by a familiar midwife (*β* = −0.43; 95% CI: −0.51 to −0.35), and perceived full support in managing pain (*β* = −0.33; 95% CI: −0.45 to −0.22).

## 4 Discussion

### 4.1 Sociodemographic Factors

Our findings demonstrated that the increase in age and education significantly widened gaps in various service quality dimensions. This could be explained by the fact that increase in mothers' age could lead to greater childbirth experience, making them more attuned to any potential quality gaps. Further, the caregivers' assumptions that older mothers are more informed and knowledgeable about birthing procedures could lead to disparities in responsiveness and support compared to young and first‐time mothers. Similar results were reported elsewhere [37].

Prior research has indicated that mothers with higher education levels maintain higher expectations regarding childbirth care due to their exposure to more birth‐related information and greater awareness of healthcare standards and practices [4, 17]. It is also possible that the increase in education may improve mothers' ability to communicate their needs, preferences, and concerns to healthcare providers.

### 4.2 Obstetric History

Our results showed that quality gaps in intrapartum care were also attributed to obstetric history aspects such as extended active labour phase (>8 h). This aligns with other studies that have emphasized the potential challenges and increased demands on healthcare providers during prolonged labour, which could impact their emotional, cognitive, and behavioural communication skills [17, 38]. Researchers argue that after long hours of labour, caregivers may undergo increased distress, burnout, and compassion fatigue, making them less capable of providing compassionate care [39].

We also observed a relationship between caesarean births and reduced gap scores in the responsiveness and assurance dimensions, along with the overall service quality. It is plausible that the surgical nature of the caesarean section (C‐section) and the associated pre‐ and postoperative care might contribute to a more structured and predictable care environment. Further, the medical necessity often associated with caesarean births could prompt healthcare providers to prioritize and execute care tasks promptly and effectively, leading to better scheduling and more efficient coordination of medical staff, resources, and facilities. There is also a prevalent belief among mothers that vaginal delivery entails more pain and anxiety, while a C‐section is perceived as a pain‐free procedure due to the use of anaesthesia [40]. Consequently, women without C‐section plans may enter the labour room with pre‐existing fears about vaginal delivery, affecting their perception of caregiver responses and reassurance. Similar results have been seen in other contexts [2, 17, 41].

A significant increase in the empathy gap was also reported for mothers who experienced PPH. Globally, PPH is one of the leading causes of maternal mortality in developing countries, and therefore, encountering such life‐threatening medical condition often compels healthcare providers to focus primarily on the urgent situation, potentially diminishing their focus on emotional support [36]. Furthermore, the high levels of anxiety experienced by caregivers during critical circumstances may also hinder their ability to clearly express their empathetic feelings toward mothers.

### 4.3 Service‐Related Factors

Our study demonstrated that multiple prior hospital admissions decreased gaps between mothers' intrapartum care expectations and perceptions. The most probable explanation could be that familiarity with the hospital environment can mitigate uncertainty and over‐expectation about future care, enabling women to form more realistic perceptions of the responsive quality compared to first time patients [8, 42]. This highlights the importance of care continuity in providing more stable and predictable quality of intrapartum care across multiple admissions [42].

Prior research has also demonstrated that having a known midwife and pre‐existing interpersonal communication can reduce mothers' sense of anxiety and stress and minimize miscommunication that might result from unfamiliar faces or frequent provider shift changes during labour [43].

Morning childbirth admission was also noted to offer mothers additional advantages in improving intrapartum care quality. Such effects can be attributed to hospital care operational patterns during daytime hours, where the availability of resources and medical personnel is often optimized, facilitating prompt attention and efficient care delivery. Moreover, mornings often mark the beginning of staff shifts, which could mean that the caregivers are well‐rested and more prepared to provide more responsive care. This scenario contrasts with nighttime or periods of shift changes, which might entail reduced staffing levels and resources [20, 43].

### 4.4 Pain Assessment and Management

In our study, women who experienced pain intensity beyond their expected levels were more prone to reporting expanded service quality gaps. It is possible that these women felt a loss of control over the birthing process and expected additional care and support to cope with their unexpected pain. This finding aligns with another observation in our study, indicating that perceiving caregiver support in childbirth pain management could significantly reduce the overall quality gap.

Several studies have underscored the vital role of caregivers' encouragement, emotional support, and expressions of affection in empowering mothers' sense of internal control and capability and making labour a less frightening and unpleasant experience [44, 45]. A recent qualitative study conducted in Jordan identified three main themes in women's perceptions of midwives' behaviour during childbirth: 1) feelings of fear, humiliation, neglect, and disrespect, citing both physical and emotional noncaring behaviours; 2) negative perceptions of midwives' conduct, including reports of disrespectful behaviour and emotional abandonment; and 3) a preference for midwives who actively listened, showed respect, and were fully present when needed [18].

Another significant factor impacting the quality of intrapartum care was the presence of a female obstetrician as the primary supervising physician during childbirth. Researchers argue that female physicians are likely to exhibit significant engagement in providing psychosocial counselling, empathetic questioning, and mutual active listening with labouring women [46, 47]. This patient‐centred approach is possibly influenced by their shared gender experiences, which grant them a deeper understanding of the physical, psychological, and emotional aspects of childbirth. Further, the cultural sensitivity involved in childbirth procedures highlights the crucial role of female physicians in providing effective and focused communication and enhancing mothers' ability to address gender‐specific concerns with their physicians [48].

## 5 Study Implications

### 5.1 Enhancing Intrapartum Care Responsiveness for Mothers

To avoid creating unrealistic expectations or misleading promises, healthcare providers should ensure that their communications with mothers accurately reflect their service features, benefits, and limitations. Overpromising and underdelivery of promised services can further widen the responsiveness gap. To mitigate this issue, caregivers should consider more personalized care and increased attentiveness towards providing prompt performance of both medical and nonmedical services throughout childbirth process. A key component to reducing mothers' feelings of anxiety and earning their trust is providing them with clear and transparent communication about the timing and execution of procedures, interventions, and other care‐related tasks. In addition, improved handover between staff shifts would help to improve the exchange of critical information between caregivers, prevent potential delays, and ensure the continuity of care.

Considering the significant impact that admission timing and history can have on the quality of intrapartum care, implementing an orientation for women in the labour ward and ensuring the availability of resources at all times can significantly promote the childbirth experience. Addressing the individual needs of all mothers, regardless of age, can also play a vital role in offering a more consistent and equitable experience.

### 5.2 Bridging the Assurance Gap in Intrapartum Care

Given that the deviation between a mother's actual pain level and her expectations can create a gap that extends beyond the physical aspect to encompass psychological and emotional needs, our study stresses the importance of effectively managing and aligning maternal pain expectations and maintaining a positive women‐provider relationship. To help achieve that, caregivers should be trained on the development of better interpersonal skills and communication strategies, and the effective utilization of appropriate pain relief options. Establishing a trusting relationship with mothers, providing them with clear explanations about birthing procedures, responding to their needs promptly, and actively engaging them in decision‐making would all help in ensuring a satisfactory overall childbirth experience.

Our findings also suggest that healthcare managers should prioritize care continuity through considering staffing patterns that promote women's access to familiar midwives and minimize changes in labour staff during childbirth. Investing in midwifery workforce development and providing support for midwifery practices will also be crucial in meeting the needs and expectations of mothers.

### 5.3 Fostering Empathic Care for a Supportive Childbirth Experience

Creating a supportive environment that ensures mothers feel heard, understood, and encouraged can alleviate pre‐existing feelings of anxiety and fear associated with childbirth, especially vaginal delivery, which have been identified as a factor contributing to an increase in requests for C‐sections. The development of empathetic skills should be one of the underlying objectives in the continuous education of healthcare professionals. Labouring women often vocalize their need for attention, and thus, caregivers should be empathetically attentive, particularly during moments of uterine contractions or when the mother senses the urge to push the baby, as she becomes increasingly vulnerable due to heightened pain. Providing empathic care can empower women, boost their confidence in their ability to manage labour pain, and enhance their overall birth experience. This would, in turn, help in promoting the likelihood of successful vaginal deliveries while reducing the requests for elective C‐sections.

Acknowledging the impact of physicians' gender on empowering childbirth experience emphasizes the importance of considering women's specific gender preferences and their choice to prioritize the empathy of the physician. Having both male and female physicians who have received training in developing empathetic communication skills is crucial for understanding the emotional needs of labouring mothers.

## 6 Study Strength and Limitations

In Jordan, much research on the quality of intrapartum care and its potential measurement has relied on routine data sources that are not designed for quality assessment, while others mainly focus on evaluating women's general satisfaction with the provided services. The strength of this study lies in its utilization of a standardized SERVQUAL tool, wherein the validity and reliability of scale have been thoroughly examined to be confidently asserted. Employing the SERVQUAL scale also allows for a comprehensive assessment of quality care across multiple quality dimensions. Further, the use of a facility‐based pre–post design provides a strong foundation for assessing intrapartum care quality pre‐ and postchildbirth, allowing for direct comparisons.

However, this study is not without limitations. Participants were selected using a convenience sampling strategy from a single university hospital; therefore, it should be noted that the generalizability of our findings to other facilities is limited. Future studies are recommended to include mothers from both public and private healthcare facilities to explore differences in quality gaps between these settings. Labour is a multistage process, and therefore the evaluation of variations in the quality of intrapartum care during each phase of the childbirth process needs to be further investigated.

## 7 Conclusion

The overall perception scores of the service quality were lower than the corresponding expectation scores for all SERVQUAL dimensions. However, significant negative quality gaps were identified in the dimensions of assurance, empathy, and responsiveness, as well as overall service quality. Our findings underscore the importance of identifying and understanding the factors that contribute to the gaps between mothers' expectations and perceptions of intrapartum care quality, including education, birth mode, admission timing, active labour duration, continuity of midwifery care, physician's gender, and pain management during labour. This would help healthcare providers, managers, and policy makers in taking proactive measures and implementing appropriate strategies to improve the birthing experience. Fostering a labouring environment that prioritizes the enhancement of caregivers' empathetic, communicative, and responsive skills is essential to minimizing service quality gaps and enhancing the overall childbirth experience for women in Jordan.

## Author Contributions

**Heba Hijazi:** conceptualization, methodology, validation, formal analysis, writing–review and editing, writing–original draft, funding acquisition, investigation. **Nabeel Al‐Yateem:** conceptualization, methodology, writing–original draft, writing–review and editing. **Rabah Al abdi:** conceptualization, writing–original draft, writing–review and editing, formal analysis. **Wegdan Baniissa:** conceptualization, writing–original draft, writing–review and editing, methodology. **Mohamad Alameddine:** conceptualization, writing–original draft, writing–review and editing, methodology. **Alham Al‐Sharman:** conceptualization, writing–original draft, writing–review and editing, methodology. **Alounoud AlMarzooqi:** conceptualization, writing–original draft, writing–review and editing. **Muhammad Arsyad Subu:** conceptualization, writing–original draft, writing–review and editing. **Fatma Refaat Ahmed:** conceptualization, writing–original draft, writing–review and editing. **Ahmed Hossain:** writing–original draft, formal analysis, writing–review and editing. **Amer Sindiani:** conceptualization, methodology, investigation, writing–original draft, writing–review and editing. **Yaseen Hayajneh:** writing–original draft, writing–review and editing, conceptualization.

## Conflicts of Interest

The authors declare no conflicts of interest.

## Data Availability

The data that support the findings of this study are available on request from the corresponding author. The data are not publicly available due to privacy or ethical restrictions.
